# Supplementary material for: Biological Applications of Synthetic Binders Isolated from a Conceptually New Adhiron Library
Source: Biomolecules. 2023 Oct 17;13(10):1533. doi: 10.3390/biom13101533 (PMC10605594; doi:10.3390/biom13101533)
Supplement: Supplementary file 1 [file biomolecules-13-01533-s001.zip › biomolecules-2598880-supplementary.pdf]

Supplementary Information

Figure S1. Screening of anti-pea protoplast Adhirons by flow-cytometry

Examples of flow cytometry screening results obtained using anti-pea protoplast Adhirons (B01) in combination with anti-ALFAtag nanobodies fused to mRuby3. Overlapping red emission spectra of protoplasts only (orange), protoplasts + anti-ALFAtag-mRuby3 (black line) and protoplasts + anti-ALFAtag-mRuby3 in the presence of Adhiron clone (light blue) for both a negative (left) and a positive ligand (right).

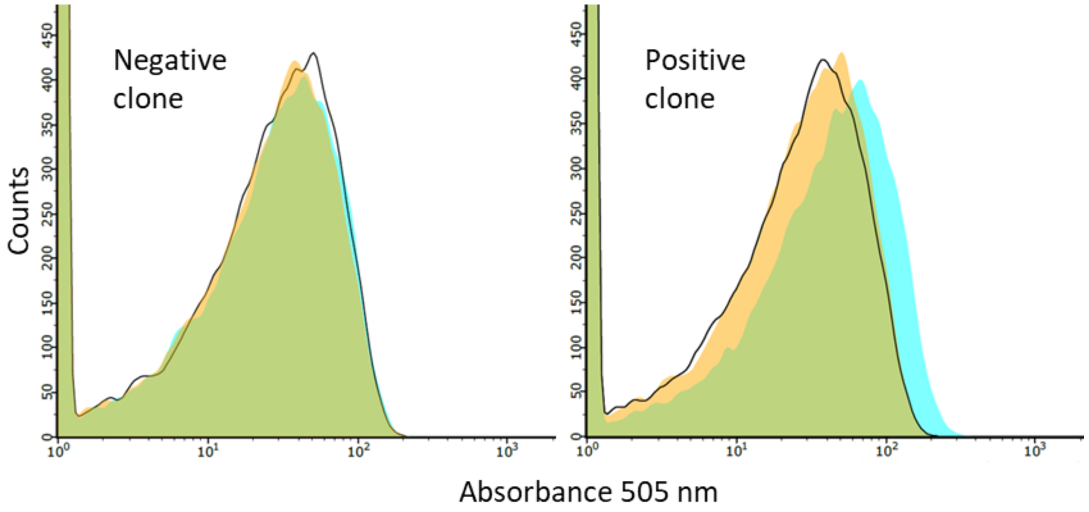

Figure S2. Unique sequences of anti-pea protoplast Adhirons

a) Each clone is represented by a code, its isoelectric point is reported, the variable regions are in green and the cysteines in red.

A12, pI 6.30
MATGVRAVPGNENSLEIEELARFAVDEHNKKENALLEFVRVVKAKEQCIRGFCGGGTMYYLTLEAKDGGKKKLYEA KVWVKHIDHYIDYGNFKELQEFKPVGDA
B01, pI 5.87
MATGVRAVPGNENSLEIEELARFAVDEHNKKENALLEFVRVVKAKEQCIGGCVGGGTMYYLTLEAKDGGKKKLYEA KVWVKRFVCDNDCGNFKELQEFKPVGDA
B02, pI 6.58
MATGVRAVPGNENSLEIEELARFAVDEHNKKENALLEFVRVVKAKEQCIGGCCGGGTMYYLTLEAKDGGKKKLYEA KVWVKRSDCRSDCCNFKELQEFKPVGDA
E12, pI 6.60
MATGVRAVPGNENSLEIEELARFAVDEHNKKENALLEFVRVVKAKEQCIGGVVGGGTMYYLTLEAKDGGKKKLYEA KVWVKRVVRDNDGGNFKELQEFKPVGDA

b) CLUSTAL O (1.2.4) multiple sequence alignment of anti-protoplast Adhiron clones

Variable sequences are in red, cysteines are highlighted in blue.

```
B01       MATGVRAVPGNENSLEIEELARFAVDEHNKKENALLEFVRVVKAKEQCIGGCVGGGTMYY     60
E12       MATGVRAVPGNENSLEIEELARFAVDEHNKKENALLEFVRVVKAKEQCIGGVVGGGTMYY     60
A12       MATGVRAVPGNENSLEIEELARFAVDEHNKKENALLEFVRVVKAKEQCIRGFCGGGTMYY     60
B02       MATGVRAVPGNENSLEIEELARFAVDEHNKKENALLEFVRVVKAKEQCIGGCCGGGTMYY     60
          *********************************************** *   *******

B01       LTLEAKDGGKKKLYEAKVWVKRFVCDNDCGNFKELQEFKPVGDA
E12       LTLEAKDGGKKKLYEAKVWVKRVVRDNDGGNFKELQEFKPVGDA
A12       LTLEAKDGGKKKLYEAKVWVKHIDHYIDYGNFKELQEFKPVGDA
B02       LTLEAKDGGKKKLYEAKVWVKRSDCRSDCCNFKELQEFKPVGDA
          *********************:       *   **************
```

Variable loops (1x1C, 1x2C, 1x4C, 1x6C)

Figure S3. Unique sequences of anti-CRP Adhiron clones

a) Each clone is represented by a code, its isoelectric point is reported, the variable regions are in green and the cysteines in red. Single framework mutations in G6 are in blue.

A9, pI 9.10

MATGVRAVPGNENSLEIEELARFAVDEHNKKENALLEFVRVVKAKEQHIGRVRNHYTMYYLTLEAKDGGKKKLYEA KVWVKSVIHYHYFRNFKELQEFKPVGDA

B5, pI 6,74

MATGVRAVPGNENSLEIEELARFAVDEHNKKENALLEFVRVVKAKEQSIGVGFVNVTMYYLTLEAKDGGKKKLYEA KVWVKYVSHCYGDRNFKELQEFKPVGDA

E5, pI 9.13

MATGVRAVPGNENSLEIEELARFAVDEHNKKENALLEFVRVVKAKEQFHFNRHGLFTMYYLTLEAKDGGKKKLYEA KVWVKPLVRHKAYWNFKELQEFKPVGDA

E7, pI 9.42

MATGVRAVPGNENSLEIEELARFAVDEHNKKENALLEFVRVVKAKEQYRSGYRCHRTMYYLTLEAKDGGKKKLYEA KVWVKPVRWRCGRQNFKELQEFKPVGDA

E8, pI 9.49

MATGVRAVPGNENSLEIEELARFAVDEHNKKENALLEFVRVVKAKEQRKLLGVRFLTMYYLTLEAKDGGKKKLYEAK VWVKTRWDGRGGKNFKELQEFKPVGDA

F5, pI 9.21

MATGVRAVPGNENSLEIEELARFAVDEHNKKENALLEFVRVVKAKEQYIRHLYGIGTMYYLTLEAKDGGKKKLYEAK VWVKAMCSGRRRVNFKELQEFKPVGDA

F11, pI 6.83

MATGVRAVPGNENSLEIEELARFAVDEHNKKENALLEFVRVVKAKEQHDFFIYCGITMYYLTLEAKDGGKKKLYEAK
VWVKHYNFYVYRSNFKELQEFKPVGDA

G6, pI 4.73

MATGVRAVPGNENSMEIEELARFAVDEHNKKENALLEFVRVVKAKEQSPDCDEVATTMYYLTLEAKDGGKKELYE
ADVWVKPGSRSGSGDNYKELLEFKPVGDVA

G8, pI 8.65

MATGVRAVPGNENSLEIEELARFAVDEHNKKENALLEFVRVVKAKEQWTPRYHECGTMYYLTLEAKDGGKKKLYE
AKVWVKRRDRYHLGSNFKELQEFKPVGDA

G9, pI 9.15

MATGVRAVPGNENSLEIEELARFAVDEHNKKENALLEFVRVVKAKEQFHFSSYSRGTMYYLTLEAKDGGKKKLYEAK
VWVKPRTWWRSGGNFKELQEFKPVGDA

b) CLUSTAL O (1.2.4) multiple sequence alignment of anti-CRP Adhiron clones

Variable sequences are in red, cysteines are highlighted in blue.

```
G6   MATGVRAVPGNENSMEIEELARFAVDEHNKKENALLEFVRVVKAKEQSPDCDEVATTMYY      60
G8   MATGVRAVPGNENSLEIEELARFAVDEHNKKENALLEFVRVVKAKEQWTPRYHECGTMYY      60
E8   MATGVRAVPGNENSLEIEELARFAVDEHNKKENALLEFVRVVKAKEQRKLLGVRFLTMYY      60
F5   MATGVRAVPGNENSLEIEELARFAVDEHNKKENALLEFVRVVKAKEQYIRHLYGIGTMYY      60
E5   MATGVRAVPGNENSLEIEELARFAVDEHNKKENALLEFVRVVKAKEQFHFNRHGLFTMYY      60
G9   MATGVRAVPGNENSLEIEELARFAVDEHNKKENALLEFVRVVKAKEQFHFSSYSRGTMYY      60
E7   MATGVRAVPGNENSLEIEELARFAVDEHNKKENALLEFVRVVKAKEQYRSGYRCHRTMYY      60
F11  MATGVRAVPGNENSLEIEELARFAVDEHNKKENALLEFVRVVKAKEQHDFFIYCGITMYY      60
A9   MATGVRAVPGNENSLEIEELARFAVDEHNKKENALLEFVRVVKAKEQHIGRVRNHYTMYY      60
B5   MATGVRAVPGNENSLEIEELARFAVDEHNKKENALLEFVRVVKAKEQSIGVGFVNVTMYY      60

G6   LTLEAKDGGKKELYEADVWVKPGSRSGSGDNYKELLEFKPVGDV
G8   LTLEAKDGGKKKLYEAKVWVKRRDRYHLGSNFKELQEFKPVGDA
E8   LTLEAKDGGKKKLYEAKVWVKTRWDGRGGKNFKELQEFKPVGDA
F5   LTLEAKDGGKKKLYEAKVWVKAMCSGRRRVNFKELQEFKPVGDA
E5   LTLEAKDGGKKKLYEAKVWVKPLVRHKAYWNFKELQEFKPVGDA
G9   LTLEAKDGGKKKLYEAKVWVKPRTWWRSGGNFKELQEFKPVGDA
E7   LTLEAKDGGKKKLYEAKVWVKPVRWRCGRQNFKELQEFKPVGDA
F11  LTLEAKDGGKKKLYEAKVWVKHYNFYVYRSNFKELQEFKPVGDA
A9   LTLEAKDGGKKKLYEAKVWVKSVIHYHYFRNFKELQEFKPVGDA
B5   LTLEAKDGGKKKLYEAKVWVKYVSHCYGDRNFKELQEFKPVGDA
```

Variable loops (4 no C, 5x1C, 1x2C)

Figure S4. CRP detection using an electrochemical impedance biosensor activated with the E7 Adhiron specific for the antigen

Top panel: Nyquist plots of the bare sensor (Au only), sensor plus CRP antigen (E7 only), and of the sensor functionalized with anti-CRP in the presence of CRP (CRP-E7) with its corresponding fit; Bottom panel: Cyclic voltammograms of bare sensor (blue), sensor plus CRP antigen (orange), and of the sensor functionalized with anti-CRP in the presence of CRP (red) at scan rate of 20 mV/s

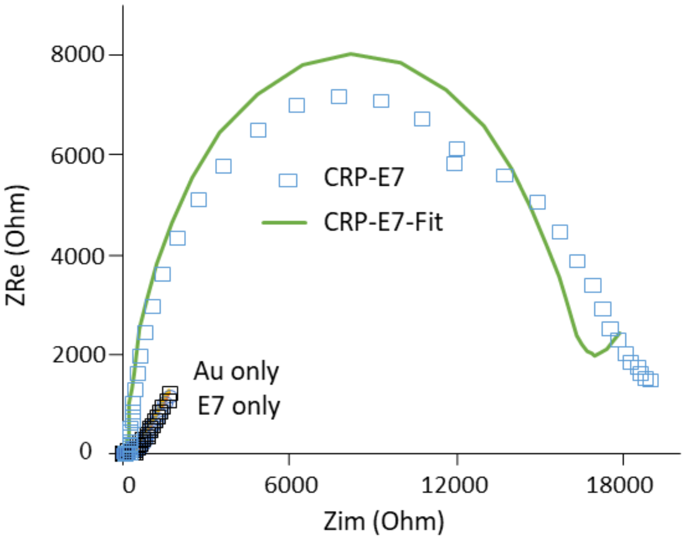

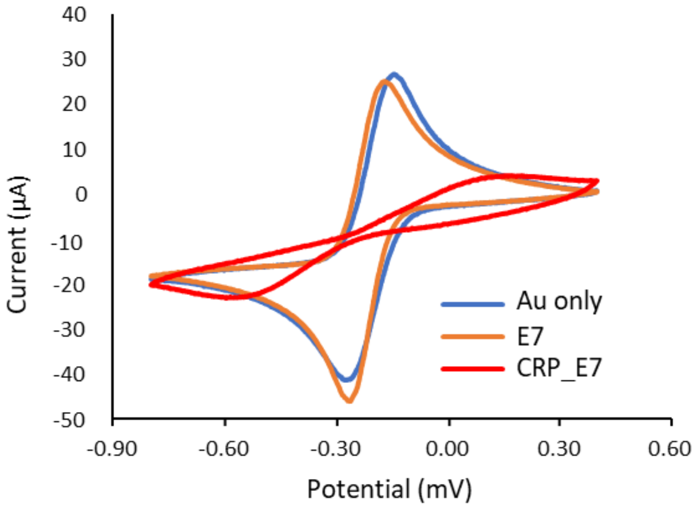

Figure S5. Unique sequences of Adhiron clones specific for SpyCatcher002

a) Each clone is represented by a code, its isoelectric point is reported, the variable regions are in green and the cysteines in red.

B1, pI 9.13

MATGVRAVPGNENSLEIEELARFAVDEHNKKENALLEFVRVVKAKEQNRRIYLSHFTMYYLTLEAKDGGKKKLYEAK
VWVKRAMPSSYFGNFKELQEFKPVGDAA

H2, pI 7.77

MATGVRAVPGNENSLEIEELARFAVDEHNKKENALLEFVRVVKAKEQLLGTTVQCMTMYYLTLEAKDGGKKKLYEA
KVWVKRICNDRHHVNFKELQEFKPVGDAA

D6, pI 7.74

MATGVRAVPGNENSLEIEELARFAVDEHNKKENALLEFVRVVKAKEQSRVIYVLWFTMYYLTLEAKDGGKKKLYEAK
VWVKHDSICCNRINFKELQEFKPVGDAA

F9, pI 5.92

MATGVRAVPGNENSLEIEELARFAVDEHNKKENALLEFVRVVKAKEQNRLIYHSDVTMYYLTLEAKDGGKKKLYEAK
VWVKHAIPDSDFGNFKELQEFKPVGDAA

G5 pI 7.74

MATGVRAVPGNENSLEIEELARFAVDEHNKKENALLEFVRVVKAKEQRVFKISGFETMYYLTLEAKDGGKKKLYEAK
VWVKHNCIYRDCFNFKELQEFKPVGDAA

B12 pI 7.74

MATGVRAVPGNENSLEIEELARFAVDEHNKKENALLEFVRVVKAKEQKDFNVFGGETMYYLTLEAKDGGKKKLYEA
KVWVKRNRICRDCHNFKELQEFKPVGDAA

b) CLUSTAL O (1.2.4) multiple sequence alignment of anti-SpyCatcher clones

Variable sequences are in red, cysteines are highlighted in blue.

```
B1      MATGVRAVPGNENSLEIEELARFAVDEHNKKENALLEFVRVVKAKEQNRRIYLSHFTMYY    60
F9      MATGVRAVPGNENSLEIEELARFAVDEHNKKENALLEFVRVVKAKEQNRLIYHSDVTMYY    60
D6      MATGVRAVPGNENSLEIEELARFAVDEHNKKENALLEFVRVVKAKEQSRVIYVLWFTMYY    60
H2      MATGVRAVPGNENSLEIEELARFAVDEHNKKENALLEFVRVVKAKEQLLGTTVQCMTMYY    60
G5      MATGVRAVPGNENSLEIEELARFAVDEHNKKENALLEFVRVVKAKEQRVFKISGFETMYY    60
B12     MATGVRAVPGNENSLEIEELARFAVDEHNKKENALLEFVRVVKAKEQKDFNVFGGETMYY    60
        ***********************************************         ****

B1      LTLEAKDGGKKKLYEAKVWVKRAMPSSYFGNFKELQEFKPVGDA
F9      LTLEAKDGGKKKLYEAKVWVKHAIPDSDFGNFKELQEFKPVGDA
D6      LTLEAKDGGKKKLYEAKVWVKHDSICCNRINFKELQEFKPVGDA
H2      LTLEAKDGGKKKLYEAKVWVKRICNDRHHVNFKELQEFKPVGDA
G5      LTLEAKDGGKKKLYEAKVWVKHNCIYRDCFNFKELQEFKPVGDA
B12     LTLEAKDGGKKKLYEAKVWVKRNRICRDCHNFKELQEFKPVGDA
        *********************         **************
```

Variable loops (2 no C, 4x2C)

Figure S6. Binding between G5-APEX and its cognate antigen SpyCatcher002 confirmed by gel filtration chromatography.

The elution profile of the G5-APEX Adhiron (double peak, grey) and of its cognate antigen SpyCatcher-mClover (single peak, peach) were compared with the profile of the complex (double peak, blue). The shift towards structures of larger mass is evident when ligand and antigen were loaded after pre-incubation.

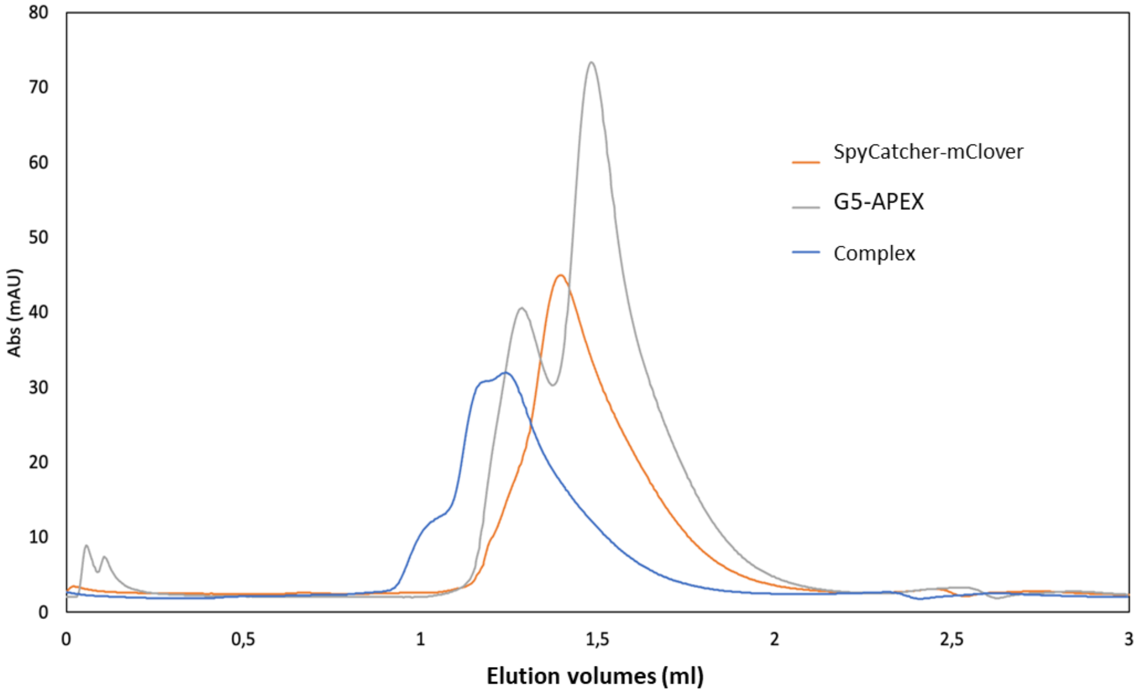

Figure S7. Affinity of G5 for SpyCatcher002 measured by chronoamperometry and fitted to Hill equation.

Affinity of G5 for SpyCatcher002 measured by chronoamperometry. Three sets of data were obtained for each of the two independent experiments, the one reported here (top panel) and the one shown in the main text (bottom panel) and the details are reported in the boxes.

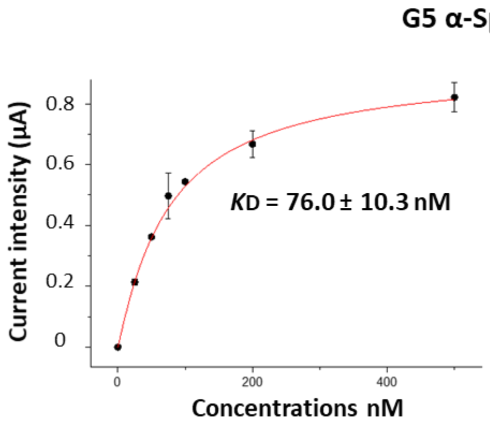

Model	Hill
Equation	y=Vmax*x^n/(k^n+x^n)
Plot	B
Vmax	9.21359E-7 ± 4.3896E-8
k	76.01593 ± 10.2817
n	1.07454 ± 0.07928
Reduced Chi-Sqr	0.10072
R-Square (COD)	0.99839
Adj. R-Square	0.99758

Model	Hill
Equation	y=Vmax*x^n/(k^n+x^n)
Plot	B
Vmax	2.36937E-6 ± 2.07047E-8
k	85.91486 ± 1.32573
n	1.13409 ± 0.02651
Reduced Chi-Sqr	2.21319E-17
R-Square (COD)	0.99998
Adj. R-Square	0.99996

Figure S8. Binding capacity of mono- and bivalent anti-SpyCatcher002 Adhiron constructs

The alternative fiber-optic-based SPR device with dip-in setting (White Fox - Fox Biosystems, Diepenbeek, Belgium) was used for comparing the binding capacity of monomeric (Top) and dimeric (via fusion to a rabbit Fc domain, Bottom) Adhiron constructs. Monovalent and bivalent Adhirons were diluted in 10 mM PBS pH 7.2 containing 0.01% Tween-20 at concentrations in the range between 216 and 1.6 nM. SpyCatcher002 was resuspended in 10 mM NaAc pH 4.5 containing 0.01% Tween-20. Data were analyzed using the manufacturer's software with a one-to-one binding model.

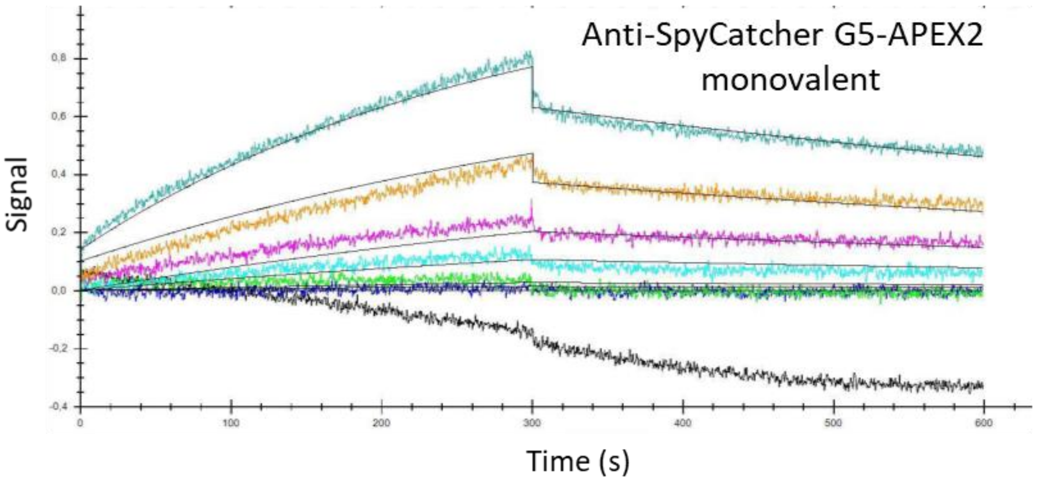

Bmax (Signal)	*k*a (1/(M*s))	*k*d (1/s)	*K*D (M)	BI (Signal)	Chi2 (Signal^2)
1.33	1.20E+04	1.05E-03	8.75E-08	0.14	0.01

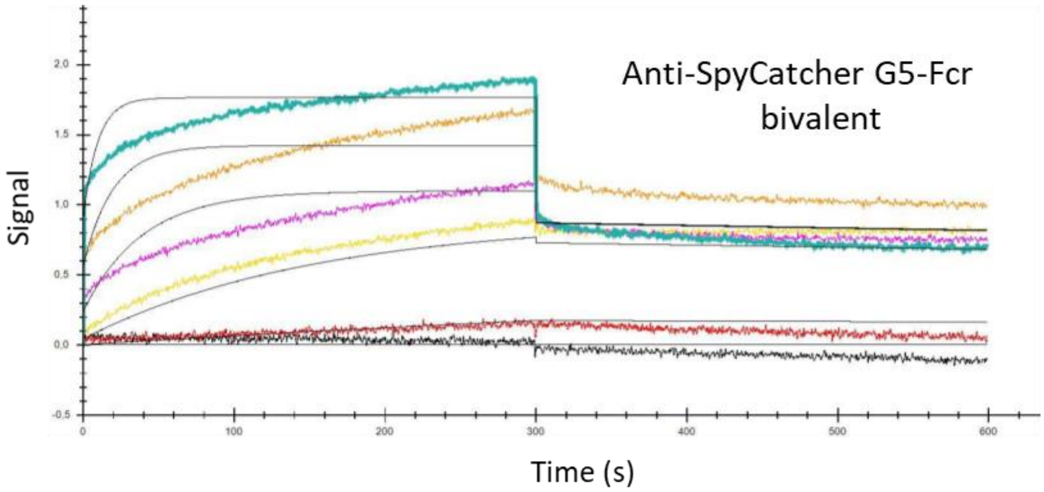

Bmax (Signal)	*k*a (1/(M*s))	*k*d (1/s)	*K*D (M)	BI (Signal)	Chi2 (Signal^2)
0.87	4.68E+05	2.16E-04	4.62E-10	0.89	0.01

Figure S9. RBD-specific Adhirons

Clone selection was performed in triplicate by comparing the specific (anti-RBD) and unspecific (anti-BSA) signals of clones chosen according to the results of the preliminary screening (single repeat). Potential candidates (absorbance >500, irrelevant background) were tested together with negative controls (C1, D8, E9, E11, G5) to evaluate the reliability of the screening method.

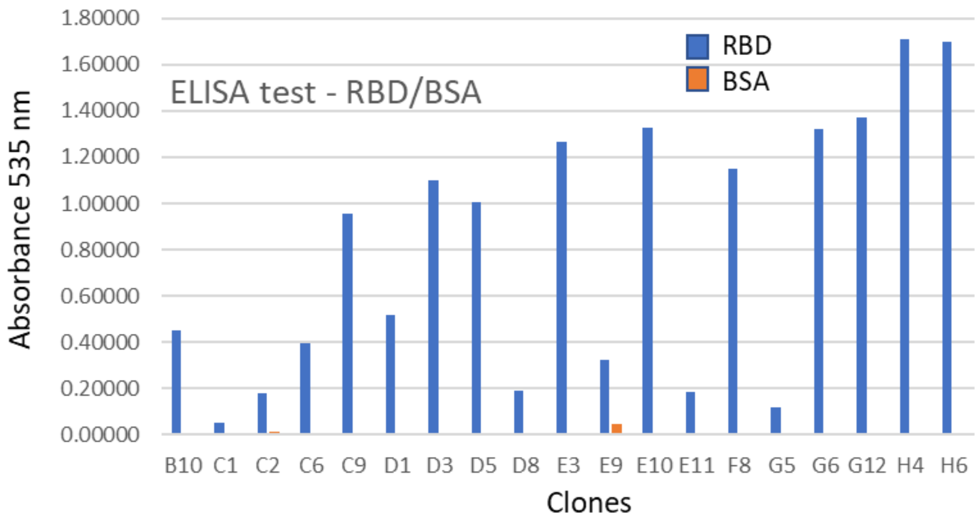

Figure S10. Heat-dependent Adhiron purification

The anti-CRP Adhiron B12-SpyTag and the Adhrion anti-SpyCatcher002 G5-cysTag were purified by inducing the precipitation of bacterial proteins via incubation of the bacterial supernatant at the indicated temperatures. Sample purity was analyzed by SDS-PAGE (top) and gel filtration (bottom). Protein mass was calculated according to the elution profile of calibration markers (Santa Cruz Biotechnology, Broad Range Markers, sc-2361).

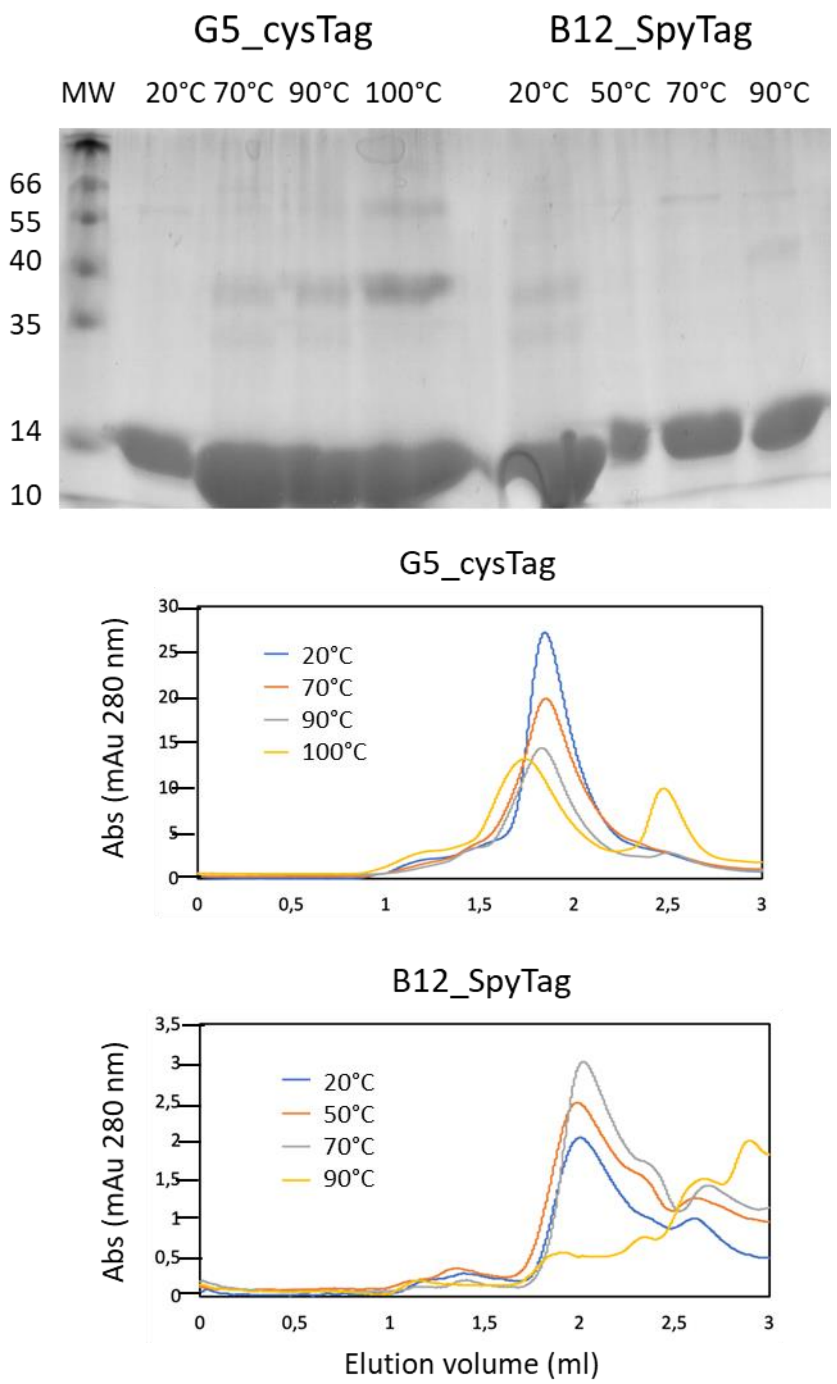
G5_cysTag
B12_SpyTag
MW
20°C 70°C 90°C 100°C
20°C 50°C 70°C 90°C
66
55
40
35
14
10
G5_cysTag
Abs (mAu 280 nm)
20°C
70°C
90°C
100°C
0 0,5 1 1,5 2 2,5 3
B12_SpyTag
Abs (mAu 280 nm)
20°C
50°C
70°C
90°C
0 0,5 1 1,5 2 2,5 3
Elution volume (ml)

Table S1. Primers used for the Adhiron library preparation

Degenerated primers were used for hypermutating the two loops. In the loop 1, X denotes NNK codons with N = A/G/C/T and K = G/T, in the loop 2, Z stands for NDT; N = A/G/C/T, D = A/G/T. These combinations enable to encode R,N,D,C,G,H,I,L,F,S,Y,V amino acids.

Primers	Sequences
Fw loop 1	5'-CAAAGCAAAAGAGCAAXXXXXXXXXXACGATGTATTATTAAC-3'
Rev loop 1	5'-CAAGGTCTGGGTAAAAXXXXXXXXXXAATTTTAAGGAACT-3'
Fw loop 2	5'-CAAAGCAAAAGAGCAAZZZZZZZZZZACGATGTATTACTTAAC-3'
Rev loop 2	5'-CAAGGTCTGGGTAAAAZZZZZZZZZZAATTTTAAGGAACTTC-3'

Table S2. Characteristics of different Adhiron-like scaffolds

Adh1 has been obtained grafting the CDR1 and CDR3 of the anti-HER2 nanobody A10 into the loops of a consensus Adhiron sequence, Adh2 grafting the CDR3 of the anti-HER2 nanobodies A10 and C8 in the same sequence, whereas in Adh3 the same sequences were cloned in an Adhiron sequence depleted of its N-terminus.

Constructs	Yield (mg/ml)	K_D (nM)
Adh1	11.6	36
Adh2	6	31
Adh3	12	0

Table S3. Unique anti-CRP Adhiron clones selected by fluorescence-based ELISA

Fluorescent signals obtained using SpyCatcher-mClover3 in combination with the SpyTag fused to Adhirons were measured at 535 nm. The results are the mean of three measurements. BSA was used for coating.

Clone	CRP	BSA
**A9**	951±49	88±7
**B5**	636±61	68±8
**E5**	1,467±89	71±4
**E7**	2,189±86	105±11
**E8**	773±27	120±9
**F5**	1,176±92	87±10
**F11**	4,960±106	185±13
**G6**	2,104±77	279±27
**G8**	4,611±211	116±6
**G9**	1,587±59	138±21

Table S4. Unique anti-SpyCatcher002 Adhiron clones selected by phage ELISA

Specific signals obtained with SpyCatcher002-mClover3 were compared with those obtained using a mClover fusion construct and the coating agent BSA. The results are the mean of three measurements.

Clones	SpyCatcher002-mClover3	A10 mClover3	BSA
B1	1,159±89	10±1	16±2
B12	580±29	17±1	10±2
D6	1,507±104	111±12	26±3
F9	1,578±53	12±2	10±1
G5	1,520±74	23±4	24±2
H2	785±49	20±1	11±1

Table S5. Buffer optimization allows the increase of the construct Tm

The construct B12-SpyTag was resuspended in different buffers and the samples underwent DSF to determine their Tm values. The highest and lowest combinations are highlighted in red.

Buffer conditions	Tm (°C)
pH 6.5	76.2
pH 6.5 + NaCl	76.4
pH 6.5 + NaCl + DTT	75.3
pH 6.5 +NaCl + EDTA	75.8
pH 7.4	78.2
pH 7.4 + NaCl	77.5
pH 7.4 + NaCl + DTT	79.8
pH 7.4 +NaCl + EDTA	77.1
pH 8.5	74.2
pH 8.5 + NaCl	74.2
pH 8.5 + NaCl + DTT	76.2
pH 8.5 +NaCl + EDTA	76.1
